# Construction of a novel cuproptosis-related gene signature for predicting microenvironment, prognosis and therapeutic response in cervical cancer

**DOI:** 10.3389/fonc.2025.1532772

**Published:** 2025-10-10

**Authors:** Yaqi Cui, Zihan Liu, Lingling Zhang, Kang Men, Meng Wang, Yingxin Hu, Zhiyuan Zhang, Xianbin Liu, Xiao Wang

**Affiliations:** ^1^ Department of Pathology, School of Basic Medical Sciences and Qilu Hospital, Shandong University, Jinan, Shandong, China; ^2^ Department of Pathology, Shandong Provincial Third Hospital, Jinan, Shandong, China; ^3^ Department of Radiation Oncology, Qilu Hospital, Cheeloo College of Medicine, Shandong University, Jinan, China; ^4^ Department of General Surgery, People’s Hospital of Rizhao, Rizhao, Shandong, China

**Keywords:** cuproptosis, tumor microenvironment, prognosis, therapeutic response, cervical cancer

## Abstract

**Introduction:**

Cervical cancer is a common malignant tumor in females, and its carcinogenesis needs further elucidation. Cuproptosis is a novel mode of cell death and its role in cervical cancer is largely unknown.

**Methods:**

The data of 334 cases of cervical cancer patients were extracted from public databases, including TCGA, GEO, GSCA and Msigdb databases. The R package, Kaplan-Meier and Cox regression analysis were used to construct the prediction model. To confirm the validation of the model, FOXJ1 was over-expressed in HeLa and SiHa cells. CCK-8, EdU, colony formation and Transwell assays were used to test the proliferation, invasion and migration abilities. Western blotting was utilized to examine the changes of protein levels.

**Results:**

We constructed a six-gene signature based on cuproptosis-related genes (CRGs) using consensus clustering analysis which further classified the patients into Cluster A and Cluster B. Kaplan-Meier survival analysis revealed that the prognosis of patients with cervical cancer in Cluster B was significantly better than in Cluster A (p=0.027). By analyzing the differentially expressed genes (DEGs), we optimized the subclassification as high and low DEG score types, and revealed their differences in prognosis, copy number variation and single nucleotide variation. The scoring model showed effectiveness in distinguishing prognosis, tumor staging, immune microenvironment, immunotherapy and chemotherapy sensitivity. Moreover, the overexpression of FOXJ1 (one of the DEGs) significantly decreased the proliferation, invasion, migration and Epithelial-Mesenchymal Transition (EMT) process in cervical cancer cells. FOXJ1 promoted cuproptosis in cervical cancer cells, thereby inhibiting their proliferation, migration, and invasion capabilities.

**Conclusion:**

Our study sheds light on the role of cuproptosis in carcinogenesis and is expected to facilitate the development of personalized treatment for cervical cancer.

## 1 Introduction

The burden of women specific cancers highlights the urgent need to study the underlying mechanisms for targeted interventions (1). Cervical cancer is the fourth leading cause of global female mortality, and HPV infection is the initiating factor (2). Although the emergence and promotion of the HPV vaccine had reduced the incidence rate of cancer (3), it has no effect on infected patients. In addition, patients diagnosed at an advanced stage, with a high rate of recurrence and metastasis, exhibit a poor prognosis, and even tolerance to chemotherapy and immunotherapy, presenting a challenge in cancer treatment (4, 5). The molecular mechanisms underlying the occurrence and development of cervical cancer require further elucidation. In recent years, with the application of bioinformatics technology in cancer genetics research, tumor scoring models have been investigated to guide patient risk stratification and personalized treatment; however, research in cervical cancer has still been limited (6–10). Therefore, exploring molecular models related to the response and prognosis of cancer patients was of great significance in cervical cancer.

Copper ions, as essential trace elements for the normal growth and development of the human body, are widely involved in various physiological processes of the body (11). Abnormal copper metabolism leads to cytotoxic stress and death (12). Tsvetkov et al. first proposed cuproptosis, a novel type of cell death, which differed from apoptosis, ferroptosis, and autophagy (13). During the process of cuproptosis, abnormal accumulation of copper ions within cells directly bind to the acylated components in the tricarboxylic acid (TCA) cycle, and aggregation of these mitochondrial lipoproteins and subsequent loss of iron sulfur cluster proteins induce protein toxicity and stress, leading to cuproptosis (13, 14). Therefore, cuproptosis is associated with the accumulation of intracellular copper ions and mitochondrial metabolism. Cuproptosis is also involved in the emergence and development of various malignant tumors, and cuproptosis-related genes (CRGs) are closely related to pathological staging and prediction of prognosis of tumor patients. For example, a cuproptosis-related risk model presents a good prognostic predictive value for patients with clear renal cell carcinoma (15), hepatocellular carcinoma (16), mesothelioma (17), and gastric adenocarcinoma (18). Furthermore, the cuproptosis scoring system is closely associated with the immune microenvironment of the tumor, such as infiltration levels of B cells, neutrophils, and mast cells (17). To date, research on cuproptosis and CRGs is still limited for cervical cancer.

In this study, we stratified patients based on the expression of cuproptosis-related genes in cervical cancer from TCGA and GEO databases. The results indicated that models based on cuproptosis-related genes and their differential expression gene scores can effectively predict the clinical stage, prognosis, tumor immune microenvironment, genetic variations, and drug sensitivity in patients. Consistently, the overexpression of FOXJ1 (one of the 11 DEGs) significantly decreased the proliferation, invasion, migration and EMT process in cervical cancer cells. Overexpression of FOXJ1 promoted cuproptosis in cervical cancer cells and further inhibited their proliferation, migration, and invasion. Our study highlighted the importance of cuproptosis in the carcinogenesis of cervical cancer and provided guidance for the development of personalized therapy for patients with cervical cancer.

## 2 Materials and methods

### 2.1 Data resources

Medical records and clinical data including survival data and nucleotide variation data were extracted from The Cancer Genome Atlas (TCGA) database based on the UCSC-XENA platform (https://xenabrowser.net/datapages/?hub=https://tcga.xenahubs.net:443). Clinical data from patients with cervical squamous cell carcinoma (CESC) were recovered from the Gene Expression Omnibus (GEO) database (http://www.ncbi.nlm.nih.gov/geo/) (GEO ID: GSE63514, GSE135222, GSE176307). We had uploaded all the R codes to Github (https://github.com/yushichou/Construction-of-a-novel-cuproptosis-related-gene-.git).

### 2.2 Processing of data related to cuproptosis

Analyses were conducted in R version 4.2.2. Gene Set Variation Analysis (GSVA) was conducted using software package version 1.44.0. The TCGA database was queried in January 2023. Patient survival data was obtained by merging cancer data from TCGA and GEO databases (GSE63514 dataset). Batch effect analysis was constructed using the R packages “limma” and “sva”, and the corresponding principal component analysis (PCA) was provided. A correlation analysis was used to show the correlation between 48 genes related to cuproptosis. Kaplan–Meier analysis was used to test the correlation between genes and the overall survival rate of the patients. Furthermore, protein-protein interaction networks (PPI) were drawn to display the relationships between these genes and the results of the univariate regression analysis.

### 2.3 Construction of a cuproptosis-related prognostic signature

To establish a predictive model for the prognosis of cervical cancer, a univariate Cox regression analysis was performed to identify CRGs with prognostic value. A *P-*value <0.05 was considered statistically significant. To build a prognostic signature, the R package ‘Consensus Cluster Plus’ was used to perform consensus unsupervised clustering according to the expression level of CRGs. The PCA was used for the optimization of the grouping approach based on differentially expressed genes (DEGs) in cuproptosis gene clusters. In detail, the differentially expressed gene score (DEG score) was calculated according to the formula ‘DEG score = (PC1 + PC2)’. The optimal cutoff value for stratifying patients into “high” and “low” DEG score groups was determined using maximally selected rank statistics, which identifies the score threshold that yields the maximum separation of survival curves based on the log-rank test. To assess the robustness of the prognostic scoring model based on principal component analysis (PCA), we performed bootstrap internal validation through 100 resampling iterations. In each iteration, we randomly selected 60% of the samples from the merged cohort of TCGA and GSE63514 as the training set and constructed a multivariable Cox proportional hazards model incorporating PCA scores, FIGO staging, tumor grade, and new tumor event status. Subsequently, we evaluated the model using time-dependent ROC analysis at 1-year, 3-year, and 5-year time points on the remaining 40% of samples (validation set). Finally, we calculated the Area Under the Curve (AUC) value for each iteration and determined the mean AUC and 95% CI for all iterations. We further performed multivariate Cox regression analysis in the entire cohort to assess the independent prognostic value of each factor. Kaplan–Meier analysis was conducted to compare the differences in survival rates between subgroups and a heatmap was constructed to show the clinical characteristics. The predictive ability of the established prognostic model in evaluating immune responses was validated using different cancer profiles.

### 2.4 Analysis of gene variants

Single nucleotide variant (SNV) and copy number variation (CNV) data were obtained from the Gene Set Cancer Analysis (GSCA) database (https://guolab.wchscu.cn/GSCA/#/). The mutation annotation format (MAF) was established using the ‘MAF Tools’ in the R software package, and the Oncoplot was drawn according to the descending order of mutations.

### 2.5 Enrichment pathway analysis

Kyoto Encyclopedia of Genes and Genomes (KEGG) pathway data were obtained from the Msigdb database, including the HALLMARK pathway, the KEGG pathway, and the Reactome pathway (https://www.gsea-msigdb.org/gsea/msigdb). The R “GSVA” package was used for scoring of pathways and comparing differences between types. The heatmaps were constructed using the package R ‘pheatmap’ to show the relationship between clinical characteristics, gene expression, prognosis, and typing. Gene Ontology (GO) data were collected from the Msigdb database using the R package cluster profiler and analyzed according to biological process, molecular function, and cellular component.

### 2.6 Immune and clinical analysis based on DEG scores

To test the prognostic value of DEG scores, the immunological and clinical analyses were conducted. To reveal the relationship between scores and clinicopathological characteristics, percentile bar charts were plotted presenting the ratios of different clinical stages to scores. An alluvial diagram was used to demonstrate the relationship between two subgroups and clinical staging. Additionally, the R package ESTIMATE was used to assess immune status and stromal scores, as well as their sum, and the discrepancy between different groups was compared. Furthermore, the fraction of immune cell infiltration was calculated using the single sample Gene Set Enrichment Analysis (ssGSEA) function of the GSVA R package GSVA. Subsequently, the expression of chemokines and their receptors was analyzed under different DEG scores. To explore the relationship between score and biological function, a correlation map between scoring and 50 hallmark pathways was drawn. Finally, the expression of immune checkpoints in the subgroups was displayed.

### 2.7 Prediction of drug sensitivity in nonimmunotherapy

We integrate Checkmate (KIRC) data for renal cell carcinoma with the data from a previous study (PMID: 32472114), and then analyzed the relationship between scores and response to treatment to nivolumab (anti-PD-1) versus treatment with an mTOR inhibitor, as well as their relationship with the patient prognosis. Drug sensitivity differences were compared between the high and low score groups. The “pRRophetic” package of R packages “pRRophetic” was used to calculate the half-maximum inhibitory concentration (IC50) values of multiple anticancer drugs in patients with patients with cervical cancer.

### 2.8 Cell culture and transfection

Human cervical cancer cell lines HeLa and SiHa were purchased from the American Type Culture Collection (Manassas, VA, USA). SiHa cells were cultured in RPMI-1640 medium (Gibco, Shanghai, China) and HeLa cells were cultured in Dulbecco’s modified Eagle medium (Gibco, Shanghai, China) at 37 °C with 5% CO_2_. All medium was supplemented with 10% fetal bovine serum (Gibco BRL, Grand Island, NY, USA) and 1% penicillin-streptomycin. Plasmid transfection was performed as previously described (18). Full-length FOXJ1 cDNA and vector pENTER were purchased from Vigene Biosciences (Shandong, China). The pENTER vector was used as a negative control. Ammonium tetrathiomolybdate (TTM) was purchased from AbMole (CAS No.:15060-55-6). Cells were treated with TTM at a final concentration of 40μM for 2 hours.

### 2.9 Cell proliferation assay

Cell proliferation ability was assessed using the EdU Cell Proliferation Kit with Alexa Fluor 488 (CellorLab, ShangHai, China) and the Cell-Counting Kit (CCK-8) (Abbkine, U.S.). For the EdU assay, cells were seeded into wells of 96-well plates at a density of 1×10^4^ cells per well. After EdU labeling, cells were treated with 50 µL of Click reaction solution and 50 µL of Hoechst 33342 separately and then observed under fluorescence microscopy (Olympus, Japan). The proliferation rate was determined according to the percentage of EdU-positive cells. For the CCK-8 assay, cells were seeded into wells of 96-well plates at a density of 3×10^3^ cells per well and treated according to the manufacturer’s instructions.

### 2.10 Colony formation assay

Colony formation was measured as previously described (19). In brief, cells were seeded into wells of six-well plates at a density of 500 cells per well after 48 h of transfection. After 15 days of incubation, colonies were stained with Crystal Violet Ammonium Oxalate Solution (Solarbio, China) and counted.

### 2.11 Cell migration and invasion assays

Migration assays were performed using Transwell inserts (8.0 µm, 24-well format; Corning, NY, U.S.). For the invasion assay, the inserts were precoated with Matrigel matrix (BD Science, Sparks, MD, U.S.). Assays were conducted as previously described (19).

### 2.12 Western blotting analysis

Cells were lysed using radioimmunoprecipitation assay (RIPA) buffer (NCM Biotech, Soochow, China) and total protein was extracted. The BCA reagent kit (Beyotime, Shanghai, China) was used to determine the protein concentration. Western blotting analysis was performed as previously described (19). The details of the primary antibodies are listed in **Table 1**. GAPDH was used as an internal control. Protein bands were visualized using an enhanced chemiluminescence kit (NCM Biotech, Soochow, China).

**Table 1:** Information of antibodies used in present study.

Antibody	Catalog number	WB	IHC	Specificity	Company
FOXJ1	sc-53139	1:200	1:100	Mouse polyclonal	Santa Cruz, USA
GAPDH	10494-1-AP	1:5000		Rabbit polyclonal	Proteintech, China
Vimentin	10366-1-AP	1:2000		Rabbit polyclonal	Proteintech, China
N-cadherin	22018-1-AP	1:5000		Rabbit polyclonal	Proteintech, China
Snail 1	sc-271977	1:1000		Mouse polyclonal	Santa Cruz, USA
CD19	ZM-0038		Ready to use	Rabbit monoclonal	ZSGB-BIO, China

### 2.13 Statistical analysis

GraphPad Prism v.8.0 was used for statistical analysis. Analysis of cell biological behavior with plasmid treatment involved a Student t test with two paired t-tailed or 2-way ANOVA for multiple comparisons. Data are expressed as mean ± SD. *P* < 0.05 was considered statistically significant.

## 3 Results

### 3.1 Clustering of patients with cervical cancer based on cuproptosis-related genes

**Figure 1:**
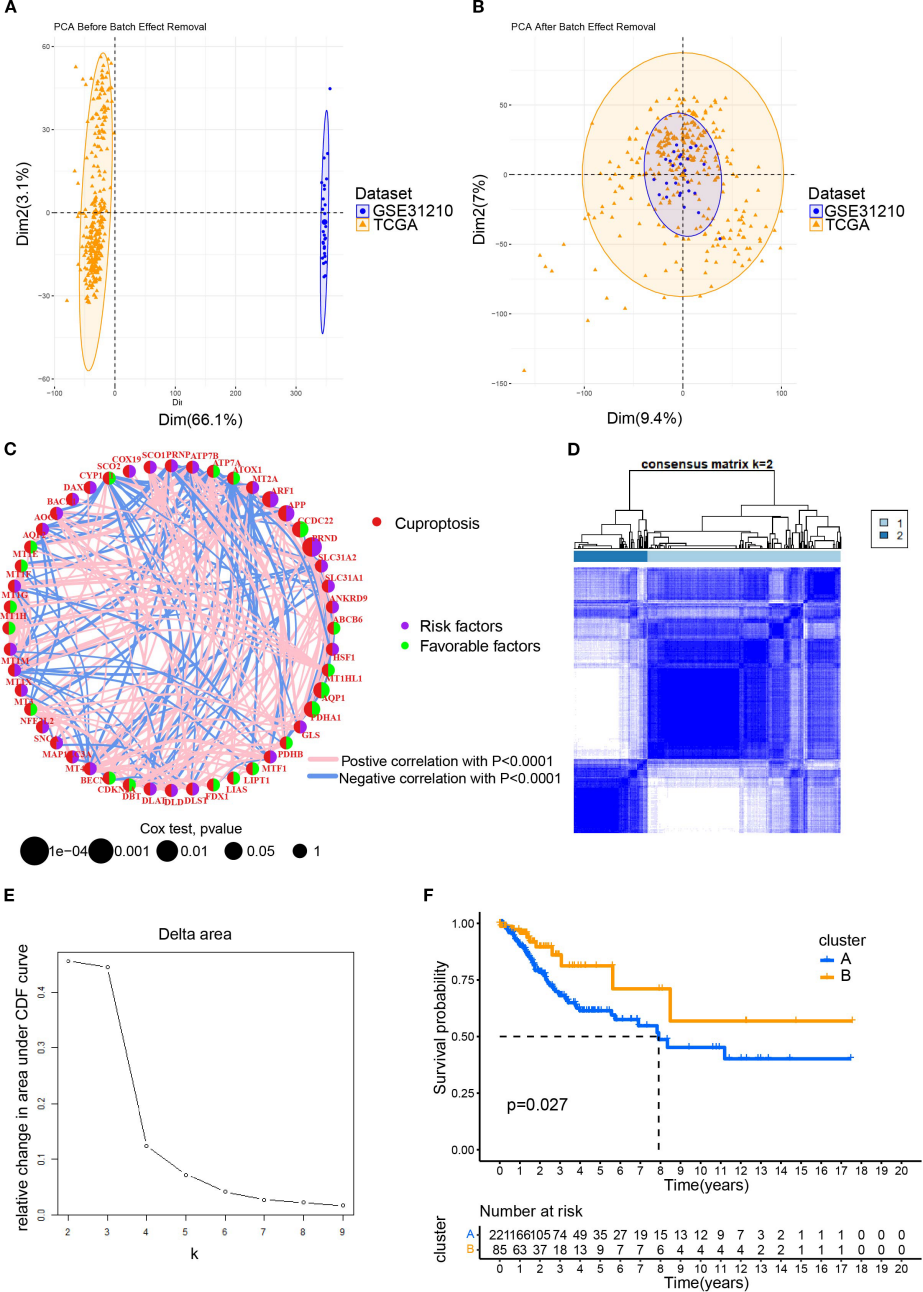
The clustering of patients with cervical cancer based on cuproptosis related genes (CRGs). **(A)** The PCA plot for the TCGA and GEO (GSE63514) datasets; **(B)** After merging the data of TCGA and GSE63514, the PCA plot was drawn using the R package “FactoMineR” and “factoextra”. Limma and sva in the R package were used to remove batch effects. **(C)** Interaction network diagram showing correlations between cuproptosis genes. Purple and green indicated prognostic risk factors and protective factors, respectively. **(D)** Unsupervised cluster typing of patients based on six genes. **(E)** Delta area plot for consensus clustering. This plot showed the relative change in the area under the cumulative distribution function (CDF) curve as the number of clusters (k) increases. **(F)** Kaplan–Meier survival analysis revealed the differences between the prognosis of patients with cervical cancer in Cluster A and B.

To elucidate the characteristics of CRGs, we merged the data of CESC in TCGA with that in the GEO database using the R package. In the batch effect adjustment, TCGA samples were assigned to batch 1, and GSE63514 samples were assigned to batch 2. Before batch correction, principal component 1 explained 66.11% of the total variance, indicating that the data structure was primarily dominated by batch effects; after batch correction, the proportion explained by PC1 decreased to 9.45%, indicating that the batch variable had been effectively removed. In total, 28 tumors in GSE63514 and 306 tumors in TCGA were enrolled, and survival data were available for 306 patients (**Figures 1A, B**). Forty-eight cuproptosis genes were selected by merging and deweighing the cuproptosis gene dataset. The relationships between genes and cuproptosis, as well as the correlation between genes, are shown in **Figure 1C**. Furthermore, we analyzed the relationship between 48 genes and the overall survival rate of the patients. Twenty-four genes were significantly correlated with the overall survival rate (**Supplementary Figure S1**). All the genes were evaluated by one-way regression analysis, and six of them (PRND, CCDC22, PDHA1, APP, ARF1, AQP1) with statistical significance were selected for subsequent analyses (data not shown). Unsupervised cluster typing showed that, based on six genes, 306 samples could be classified into two types, Cluster A and Cluster B (**Figure 1D**). The Delta Area Plot also illustrated the rationality of our grouping (**Figure 1E**). Kaplan–Meier survival analysis revealed that the prognosis of patients with cervical cancer in Cluster B was significantly better than in Cluster A (*P* = 0.027) (**Figure 1F**). The clinicopathological parameters of the patients retrieved from TCGA database were summarized in **Table 2** (those in GEO database was not available).

**Table 2:** The clinicopathological characteristics of 306 patients with cervical cancer obtained from TCGA database.

Categories	Items	Number of patients
Treatment	Radiotherapy plus Chemotherapy	139
	Chemotherapy only	6
	Radiotherapy only	38
	NA	123
Survival	Alive	74
	Deceased	232
Age (y)	> 53	96
	≤53	210
Stage	Stage I	161
	Stage II	69
	Stage III	48
	Stage IV	21
	Missing (NA)	7

The stage refers to International Federation of Gynecology and Obstetrics (FIGO).

### 3.2 Comparison of characteristics between Clusters A and B

**Figure 2:**
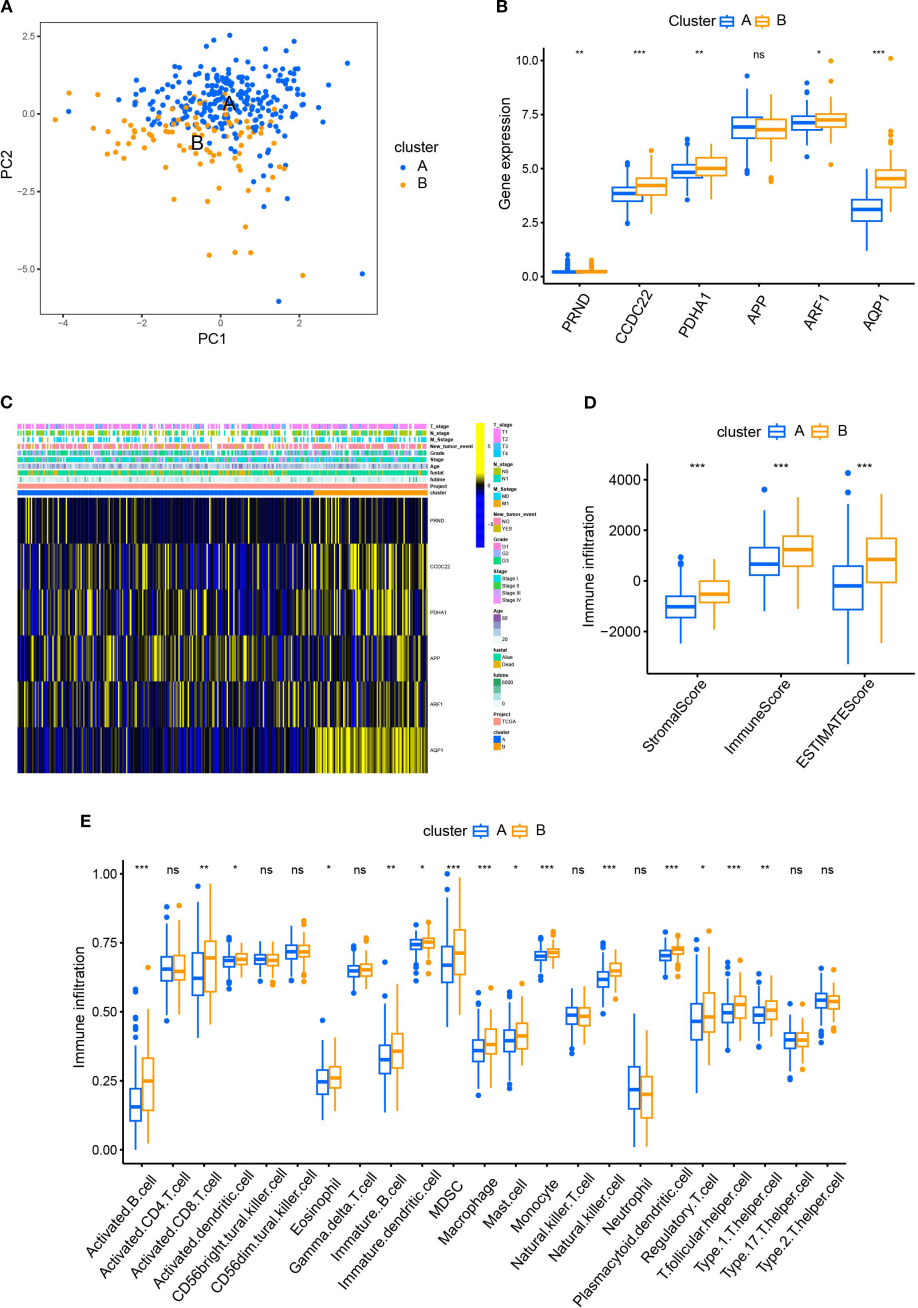
Comparison of patient characteristics between Cluster A and B. **(A)** PCA plots demonstrated the distribution of samples of Cluster A and B; **(B)** The differences of the expression between the six CRGs in Cluster A and B; **(C)** The heatmap revealed no significant difference in clinicopathological characteristics between patients of Cluster A and B. **(D)** The R package “ESTIMATE” was used to evaluate immune scores, stromal scores, and the ESTIMATE scores. The scores of Cluster B were significantly higher than those of Cluster A. **(E)** The ssGSEA function of R package GSVA was used to calculate the infiltration levels of immune cells and compare the differences between Cluster A and B.

We first demonstrated the distribution of samples of Cluster A and B using PCA plots (**Figure 2A**). Next, we compared the differences of the expression levels between the six CRGs. Except for the expression of the APP gene, the other five genes were highly expressed in Cluster B compared to Cluster A (**Figure 2B**), suggesting that the five genes could be used as potential markers to differentiate the two subtypes. However, the heatmap revealed that there were no significant differences in the clinicopathological characteristics of patients in clusters A and B (**Figure 2C**). In order to reveal the intrinsic relationship between cuproptosis and tumor immunity, we estimated the ImmuneScore and StromalScore for all samples. The score for Cluster B was significantly higher than that of Cluster A. This suggests that cuproptosis is significantly positively correlated with immune cell infiltration and stromal cell levels (**Figure 2D**). Additionally, cuproptosis was positively correlated with the tumor ESTIMATE Score, indicating a negative correlation between cuproptosis and tumor purity (**Figure 2D**). Furthermore, we calculated the levels of immune cell infiltration and analyzed their differences between Cluster A and B. As shown in **Figure 2E**, no significant differences were found between Clusters A and B in the infiltration of activated CD4+T cells, natural killer T cells, and neutrophils. However, a higher level of infiltration of activated B, CD8+T cells, dendritic cells, macrophages, and monocytes was found in Cluster B compared with Cluster A (**Figure 2E**). These results suggest a broad association between cuproptosis and tumor immunity.

### 3.2 Cuproptosis was involved in various biological processes of cervical cancer

**Figure 3:**
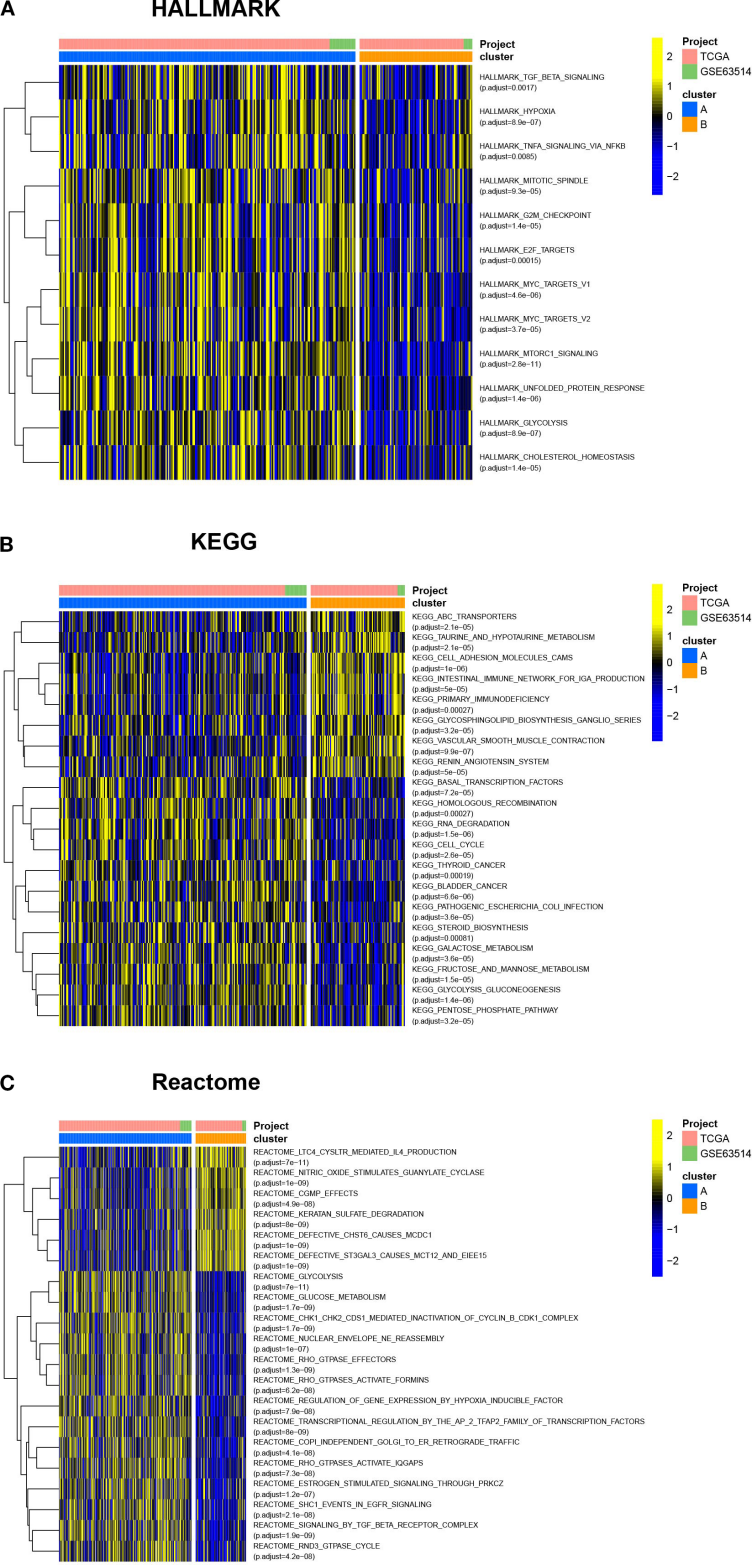
Cuproptosis is involved in various biological processes of cervical cancer. The HALLMARK pathway, KEGG pathway, and Reactome pathway were downloaded from the Msigdb database, and GSVA analysis was conducted. The heatmap showed the differences in molecular signaling pathways between Clusters A and B. ns, no significance, *p< 0.05, **p< 0.01, ***p< 0.001.

To compare the differences in molecular signaling pathways between Clusters A and B, we downloaded the HALLMARK pathway, the KEGG pathway, and the Reactome pathway from the Msigdb database and performed GSVA analysis. The results showed significant differences between Clusters A and B, indicating that cuproptosis was involved in various biological processes of cervical cancer. For example, cuproptosis was negatively correlated with hypoxia, cholesterol homeostasis, and glucose metabolism, while positively correlated with sialic acid metabolism (**Figure 3**). Furthermore, cuproptosis was negatively associated with the mitotic spindle, cell cycle, TGF-β, and TNF-α, indicating the important regulatory role of cuproptosis in tumor growth. In terms of signaling pathways, cuproptosis was associated with multiple key tumor molecules, such as E2F and MYC target genes, MTORC1, EGFR, and SMAD signaling molecules. This suggested that cuproptosis was involved in various biological processes of cervical cancer and played an important role in cervical cancer.

### 3.3 The establishment of Gene Clusters based on differential gene analysis

**Figure 4:**
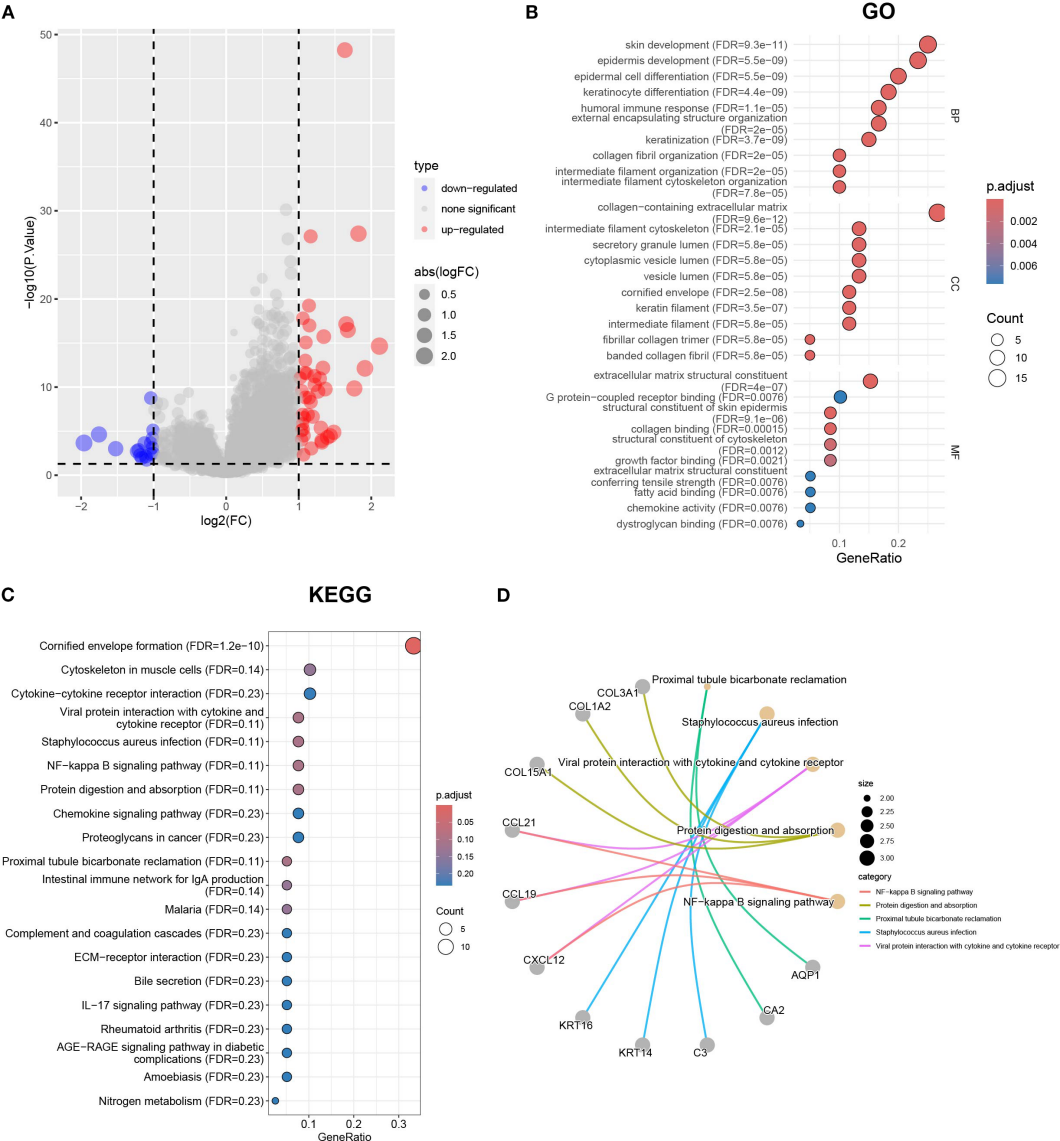
The differential gene analysis and functional annotation in Clusters A and B. **(A)** The volcano map showing the results of differential gene analysis in Clusters A and B. **(B)** Differential genes subject to GO enrichment analysis including biological processes, molecular functions, and cellular components. **(C)** KEGG enrichment analysis using |logFC|>1 and *p* < 0.05 as the threshold for differentially expressed genes. **(D)** The correspondence between the top 5 pathways and differentially expressed genes in KEGG results.

Considering that the Cluster classification method cannot predict the clinical pathological parameters of patients, we further explored the molecular background to optimize the classification. To reveal differences in gene expression profiles between Cluster A and B subtypes based on cuproptosis genes, we conducted differential analysis (**Figure 4A**). The results revealed 61 differentially expressed genes (DEGs) in Cluster A and B. To investigate the biological functions of these DEGs, we conducted an enrichment analysis using GO and KEGG. GO analysis showed that DEGs were mainly enriched in skin development, epidermal development, extracellular matrix containing collagen, and structural components of the extracellular matrix (**Figure 4B**). KEGG analysis showed that DEGs were enriched in the interaction of cytokines and cytokine receptors, the interaction of viral proteins with cytokines and cytokine receptors, and the NF-kappa B signaling pathway et al. (**Figure 4C**). The relationship between the top 5 signaling pathways and the corresponding key genes is shown in **Figure 4D**. Furthermore, we conducted a univariate regression analysis on 61 DEGs and identified 11 significantly DEGs.

**Figure 5:**
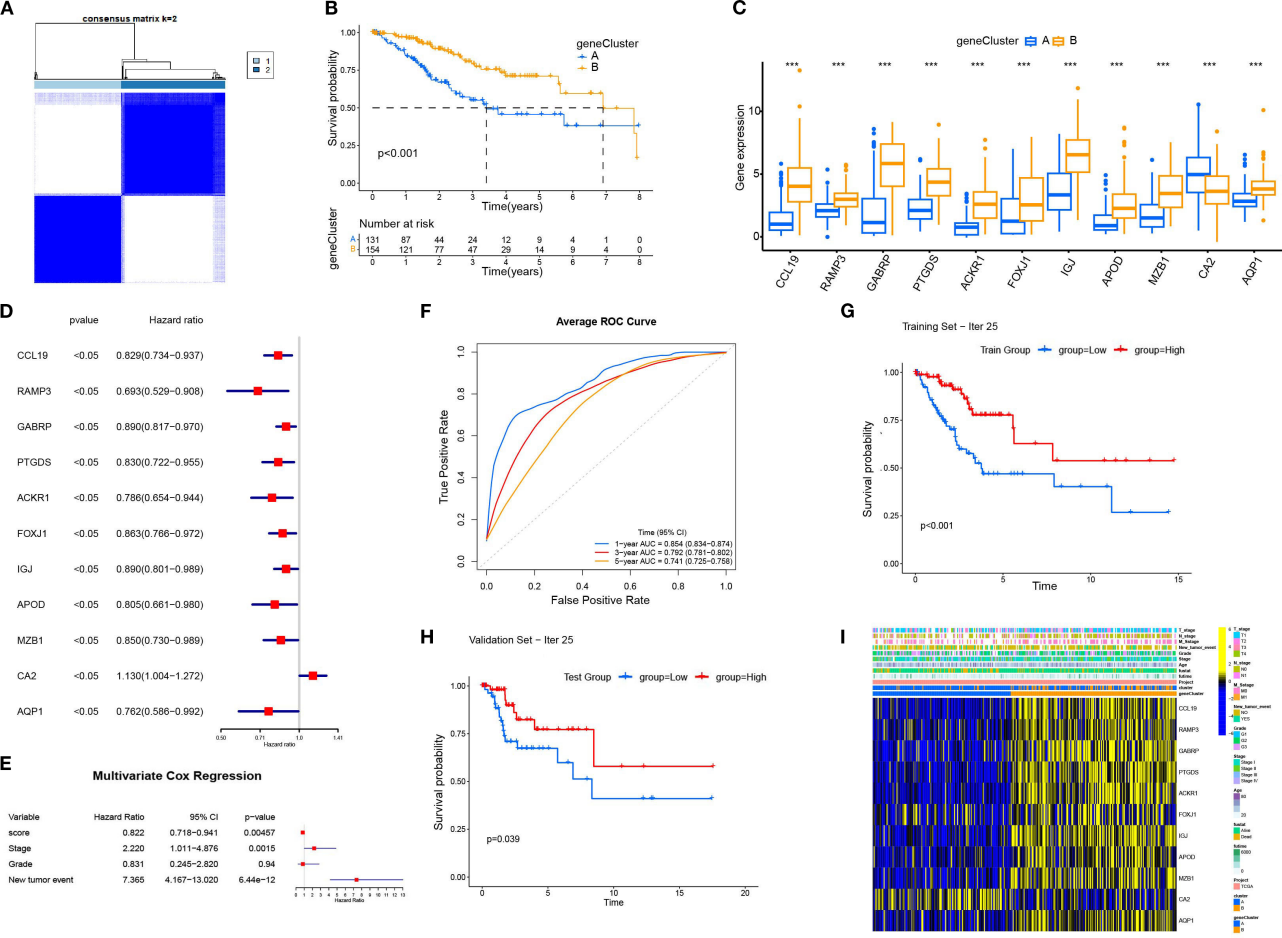
The construction and characterization of gene clustering based on differentially expressed genes (DEGs). **(A)** The consistent clustering matrix of 11 differentially expressed genes showed the best typing at k=2. **(B)** Kaplan–Meier survival curves for geneCluster A and B. **(C)** Differential expression of 11 genes in different geneClusters. **(D)** Univariate Cox regression analysis of 11 DEGs. All *p*-values <0.05. **(E)** Multivariate Cox regression analysis was performed for the clinical factors to evaluate their association with survival. **(F)** The average ROC curves for 1-year, 3-year, and 5-year survival predictions. **(G)** The Kaplan-Meier survival curve for the training set (Iteration 25). **(H)** The Kaplan-Meier survival curve for the validation set (Iteration 25). **(I)** Heatmap displaying the relationship between geneClusters and clinical pathological parameters and prognosis of patients. ***p < 0.001.

Based on the 11 DEGs (CCL19, PAMP3, GABRP, PTGDS, ACKR1, FOXJ1, IGJ, APOD, MZB1, CA2, AQP1), we constructed a cervical cancer genotyping profile to stratify patients (**Figure 5A**) and divided patients with cervical cancer into two types: gene Cluster A and gene Cluster B (**Figure 5B**). The K–M survival analysis showed that the prognosis of gene Cluster B was significantly better than that of gene Cluster A (*p* < 0. 05)(**Figure 5C**). Except for CA2, the expression of the remaining 10 genes increased in gene Cluster B, while CA2 was higher in gene Cluster A (**Figure 5D**). The heatmap showed that the gene Cluster B had an earlier T stage and clinical stage of tumor, a lower rate of lymph node metastasis and distant metastasis rate, and the survival rate of patients was significantly higher than that of the gene Cluster A (**Figure 5E**).

### 3.4 The genomic changes of cuproptosis-related DEGs in gene clusters

**Figure 6:**
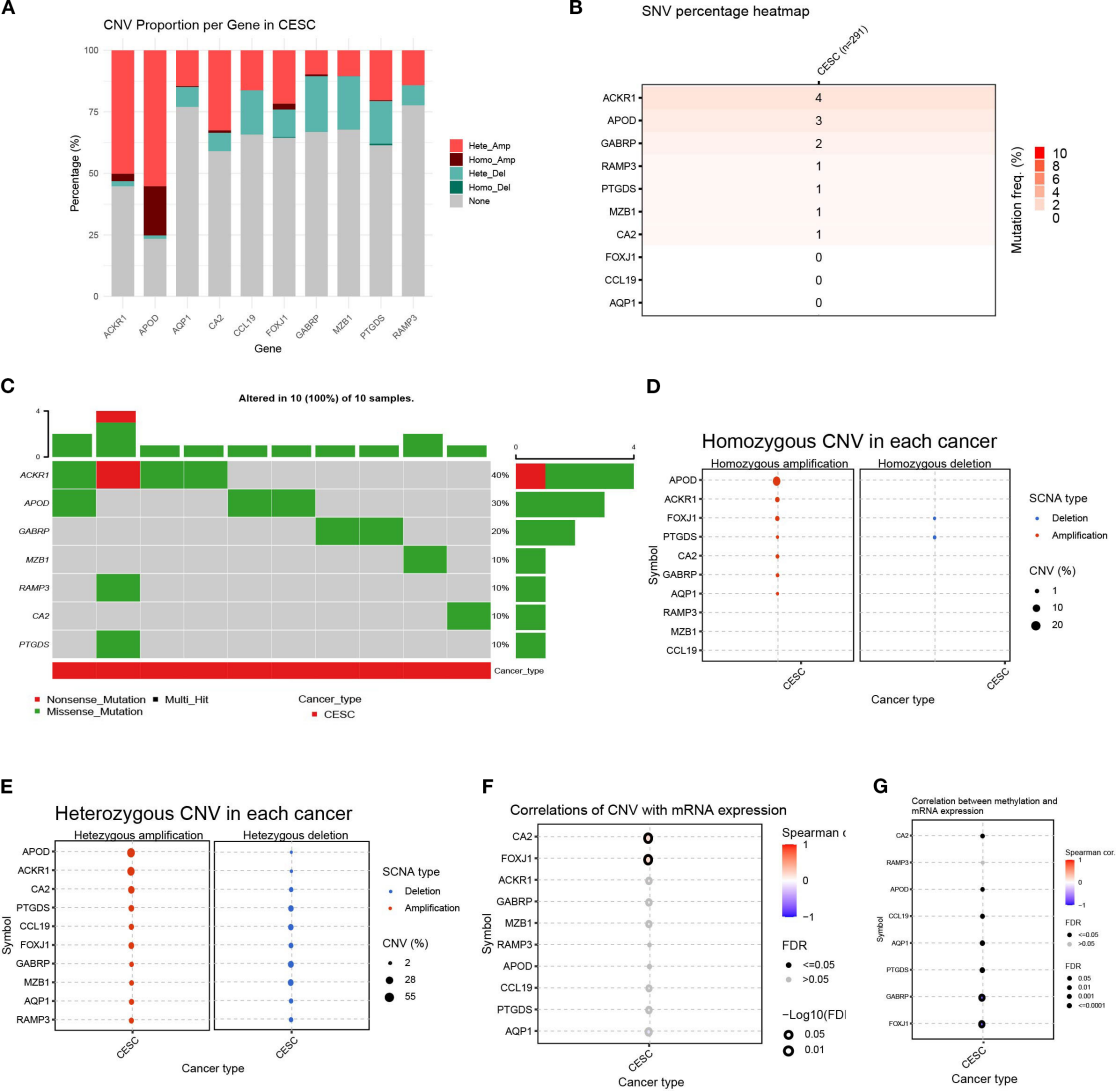
Variability of differentially expressed genes and their genetic landscape. **(A)** Proportion bar charts showing CNV types of differentially expressed genes (DEGs) in CESC. **(B)** The heatmap showing the SNV rates of DEGs in cervical cancer. **(C)** Oncoplot presented the mutation distribution of top 7 mutated genes. **(D)** The heterozygous CNV in each cancer. **(E)** The homozygous CNV in each cancer. **(F)** The correlation between CNV rates of DEGs with gene expression. **(G)** The correlation between methylation status and mRNA expression of each gene.

To better understand the genomic changes of the 11 DEGs related to cuproptosis in cervical cancer, we analyzed the single nucleotide variation (SNV) and copy number variation (CNV) data of the samples. The CNV results showed that the changes in DEGs in cervical cancer were mainly characterized by DNA amplification and deletion mutations (**Figure 6A**). The SNV results showed that the SNV frequencies of ACKR1, APOD, and GABRP were the highest (**Figure 6B**). Oncoplot analysis presented the distribution of non-sense and missense mutations of top 10 mutated genes (**Figure 6C**). The profiles of heterozygous and homozygous CNV of genes were demonstrated in CESC (**Figures 6D, E**).

Next, we analyzed the relationship between the CNV rate of cuproptosis-related genes and their mRNA expression. The results showed that the CA2 and FOXJ1 mutations were significantly positively and statistically correlated with their mRNA expression (*p* < 0. 05)(**Figure 6F**). Methylation of gene promoter regions largely negatively regulates gene expression, whereas high methylation status generally inhibits gene expression. Furthermore, we analyzed the relationship between their mRNA expression and methylation. Overall, the methylation levels of the vast majority of genes were significantly negatively correlated with mRNA levels, with GABRP and FOXJ1 being the most significant, except for the RAMP3 gene. (*p* < 0. 05)(**Figure 6G**). This indicated that the variation of the genes related to cuproptosis played an important role in the regulation of their expression in cervical cancer.

### 3.5 The relationship between DEG scores and clinical characteristics

**Figure 7:**
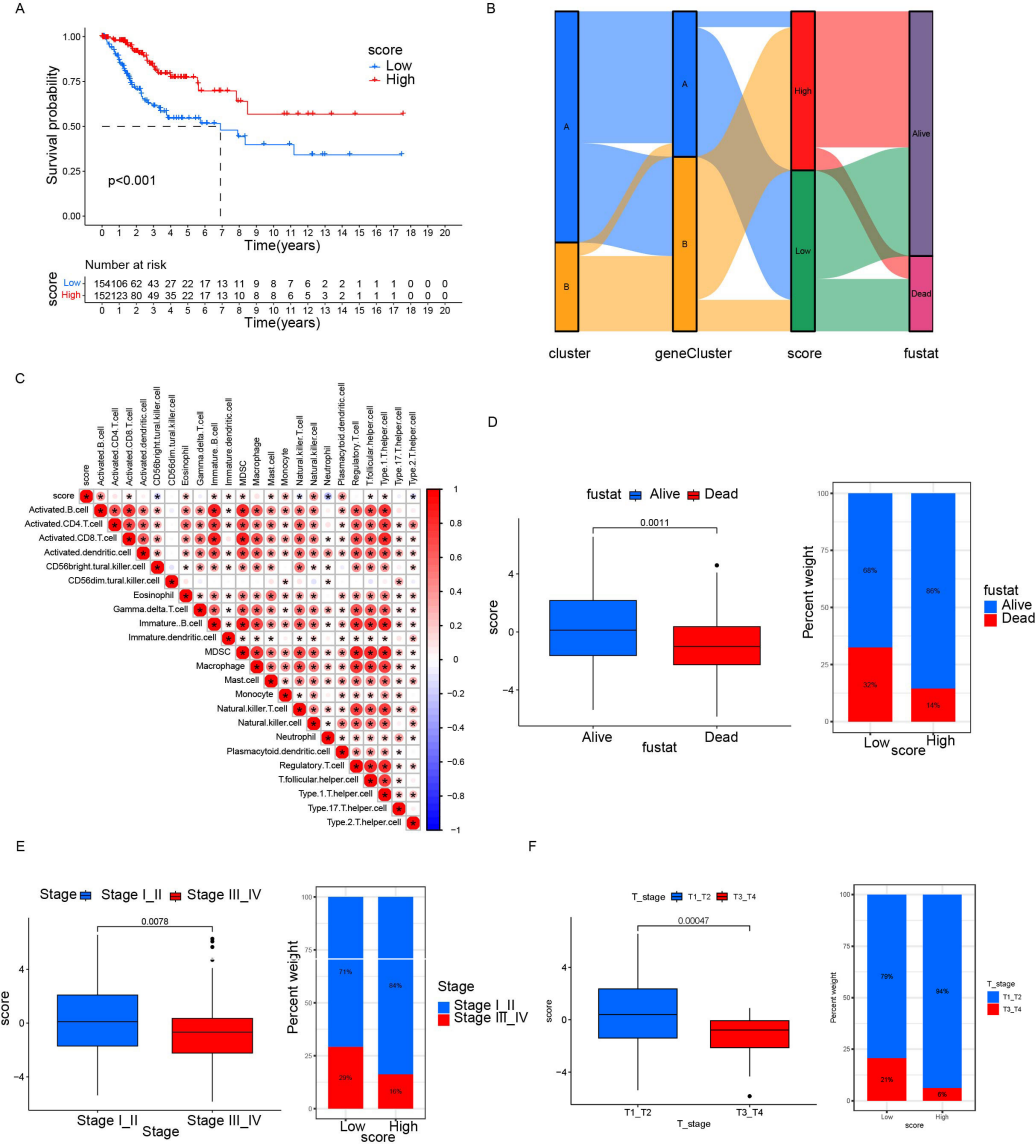
Relationship between differential gene scores and clinical features. **(A)** Kaplan–Meier survival curves stratifying patients into high and low CRG scoring groups. **(B)** Sankey diagram showing the relationship between different classification methods and prognostic status. **(C)** Correlation between score and immune cell infiltration. Red represents positive correlation; blue represents negative correlation. The darker the color, the stronger the correlation was. **(D, F)** Differences in prognosis, clinical staging, and tumor staging between groups with high and low scores. *p< 0.05.

Based on 11 DEGs, cases were scored by PCA and divided into high and low DEG score groups. To rigorously assess the reliability and robustness of the PCA score model, we performed 100 iterations of bootstrap internal validation using a merged dataset from TCGA and GSE63514. Due to the lack of external cervical cancer datasets containing complete survival information, we did not perform external validation. We fitted a multivariable Cox model incorporating PCA scores, clinical stage, tumor grade, and tumor event status, and evaluated model performance using time-dependent ROC analysis. After iteratively averaging the area under the curve (AUC) values at the 1-year, 3-year, and 5-year survival time points, the average AUC values were 0.854 (95% CI: 0.834-0.874), 0.792 (95% CI: 0.781-0.802), 0.741 (95% CI: 0.725-0.758) respectively. We plotted survival curves for the most representative bootstrap iterations (close to the average AUC), showing consistent and significant prognostic differences between the high-score and low-score groups. These results confirm that our DEG-based scoring model has good internal reproducibility and predictive value. Based on multivariate COX analysis, we plotted a forest plot showing the hazard ratio, 95% confidence interval, and *p*-value for each variable. PCA scores remained independent prognostic factors, with HR = 0.822, 95% CI = 0.718-0.941, *p* = 0.005 (**Figure 5E**). To explore the relationship between DEG scores and clinical characteristics, we established the survival analysis of patients with cervical cancer. The K–M survival analysis showed that the prognosis of patients in the high CRG score group was significantly better than that in the low DEG score group (**Figure 7A**). The Sankey plot showed the correspondence between Cluster, geneCluster, DEG score grouping, and prognostic status (**Figure 7B**). In addition, it confirmed the conclusion that the vast majority of patients with high DEG scores survived, while those who died were mostly from the low DEG score group. Furthermore, we explored the correlation between DEG scores and immune cell infiltration. The high DEG score group had a higher level of immune cell infiltration, such as activated B cells, natural killer cells, and plasmacytoid cells such as dendritic cells (*p* < 0.05) (**Figure 7C**). Furthermore, we analyzed the relationship between DEG scores and clinical characteristics (**Figures 7D–F**) and observed that high DEG scores were strongly correlated with earlier WHO staging and T1 and T2 staging, as well as higher survival rates (*p* < 0. 05). This suggested that the scoring grouping method could effectively predict the prognosis of patients. Different immune microenvironments between the high and low DEG score groups could indicate different tumor immunotherapy responses.

### 3.6 Relationship between DEG scores and tumor immune microenvironment

**Figure 8:**
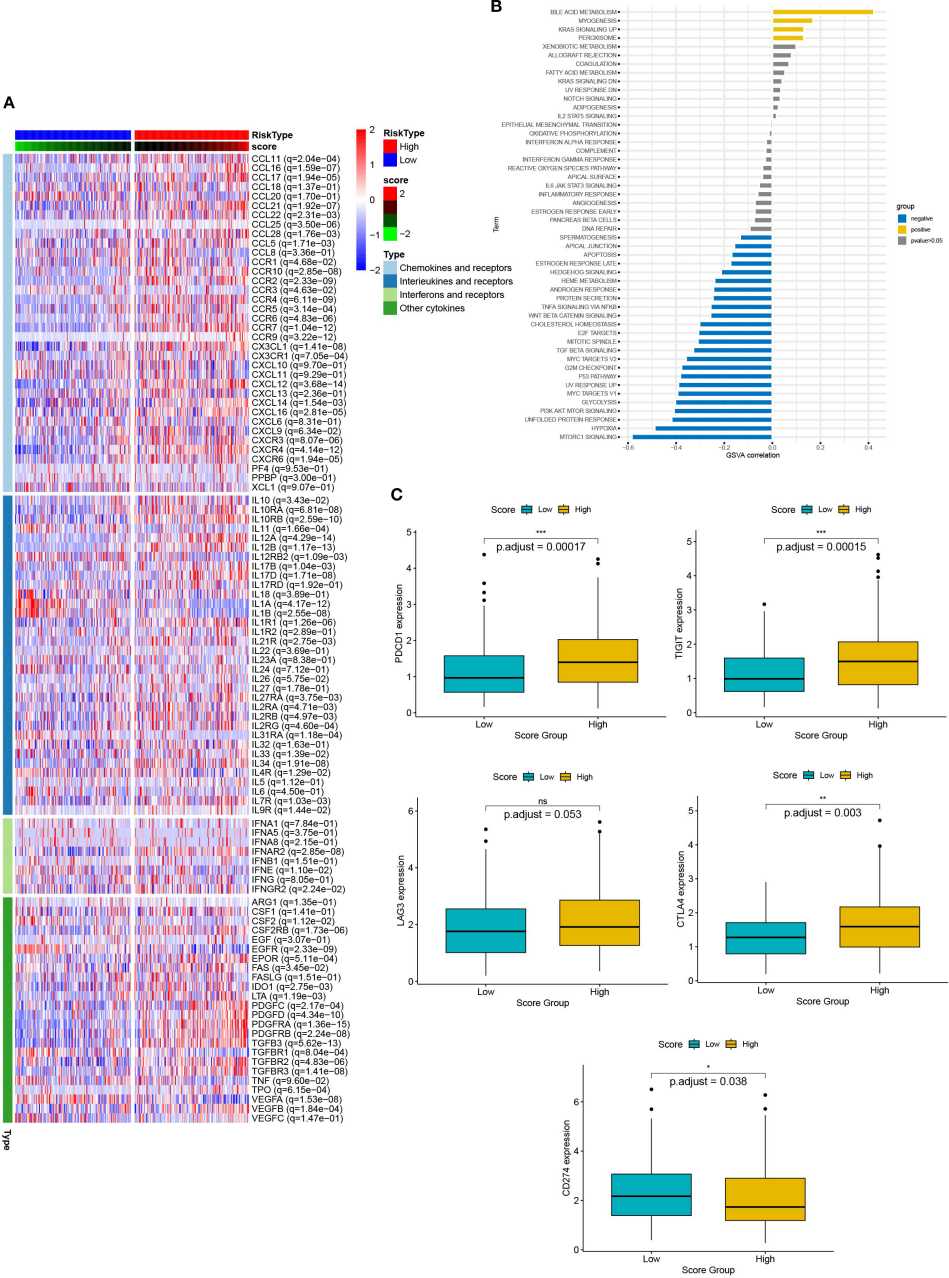
The relationship between scores and the tumor immune microenvironment. **(A)** The heat map presents the expression of chemokines and their receptors in groups with high and low scores. **(B)** Box plots showed the expression of immune checkpoints (CTLA4, PDCD1, TIGIT, and CDC274) in groups with high and low scores. **(C)** Correlation between scores and 50 hallmark pathways.

Chemokines regulated cancer cell growth and immune cell infiltration, which had a significant impact on the prognosis of the patient and the response to treatment. Therefore, we analyzed the relationship between DEG scores and expression of cytokines and receptors (**Figure 8A**). The results showed that the expression patterns of cytokines and receptors were significantly different between the high and low scoring groups. Differences in chemokines are mainly distributed in the CCL, CCR, CXCL, CXCR, and the IL families. These factors had been confirmed to be involved in immune evasion, recruitment of immunosuppressive cells, regulation of tumor angiogenesis, and metastasis. Thus, the scoring model had the potential to predict the tumor immune microenvironment and guide the patient’s prognosis and response to treatment. Additionally, we elucidated the relationship between scores and essential immune checkpoints. Compared with the low scoring group, the high scoring group had lower expression of CDC274 and higher levels of CTLA4, PDCD1, and TIGIT (**Figure 8B**). These results suggested that the cuproptosis-based scoring model could predict the expression of immune checkpoints in cervical cancer, indicating a broad correlation between cuproptosis and tumor immunity.

Next, we verified the correlation between scores and HALLMARK pathways by GSVA evaluation (**Figure 8C**). Scores were positively associated with bile acid metabolism, myogenesis, KRAS signaling up, and peroxisome pathways. In contrast, they were negatively associated with MTROC1 signaling, hypoxia, unfolded protein response, PI3K AKT MTOR signaling, p53 pathway and TGF beta signaling. Thus, multiple biological processes and pathways were correlated with scores.

### 3.7 Relationship between DEG scores and sensitivity to immunotherapy and chemotherapy in other solid tumors

**Figure 9:**
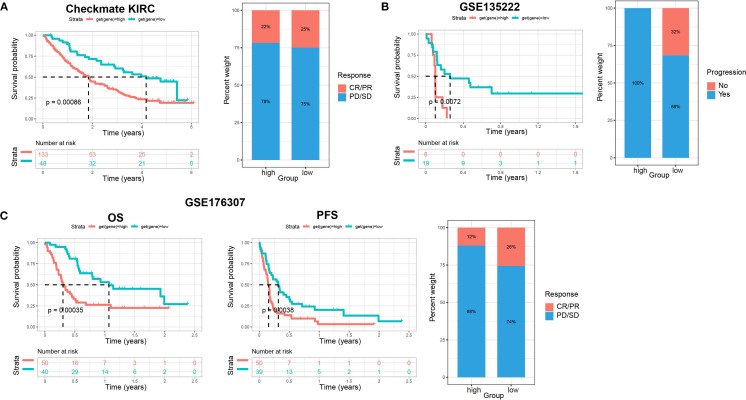
The relationship between scores and sensitivity to immunotherapy in other solid tumors. **(A)** The relationship of scores with patients’ prognosis and treatment response to nivolumab (anti PD-1) vs mTOR inhibitor in renal cell carcinoma (Checkmate KIRC). Overall survival (OS) rates on the left, and relationship between scores and immunotherapy efficacy on the right. **(B)** The relationship of scores with patient prognosis and treatment responses to anti-PD-1/PD-L1 therapy in non-small cell lung cancer (GSE135222). Progression-free survival (PFS) rates on the left, and relationship between scores and immunotherapy efficacy on the right. **(C)** The relationship of scores with patient prognosis and treatment response to immune checkpoint blockade therapy in metastatic urothelial cancer (GSE176307). OS rates on the left, PFS in the middle, and relationship between scores and immunotherapy efficacy on the right.

To further confirm the efficacy of the scoring model, we integrated data from the Checkmate (KIRC) trial of renal cell carcinoma with data from a previous study article (PMID: 32472114), and then analyzed the relationship between scores and response to treatment with nivolumab (anti PD-1) versus mTOR inhibitor, as well as their relationship with patient prognosis. Patients with low scores had significantly better survival and achieved a slightly better response to treatment (**Figure 9A**). Similar data were obtained in patients with non-small cell lung cancer who received anti-PD-1/PD-L1 therapy and with patients with metastatic urothelial cancer treated with immune checkpoint blockade therapy (**Figures 9B, C**). These findings further confirmed that patients with low scores had significantly better survival, less disease progression, and a better response to treatment. Therefore, our established prognostic scores had predictive ability for response to immunotherapy.

**Figure 10:**
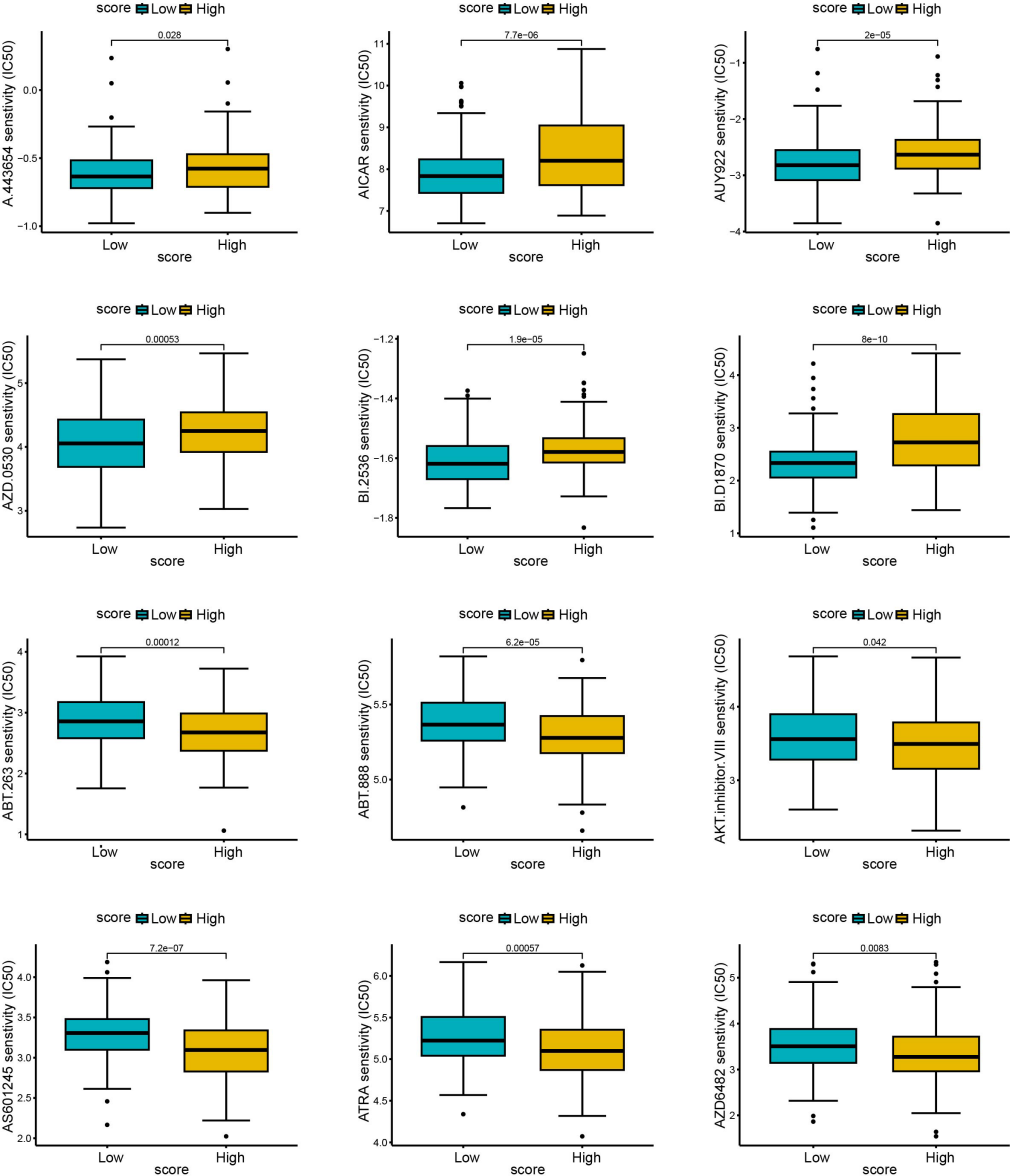
The relationship between scores and sensitivity to non-immunotherapy. The R language (pRRophetic package) was used to predict the IC50 values of each sample for multiple anticancer drugs, and the differences were compared between high and low score groups. The higher the IC50, the less sensitive the treatment was. **(A–F)**: sensitive samples; **(G–L)**: insensitive samples.

To explore the predictive outcome of this score for the response to chemotherapy in patients with cervical cancer, we performed a drug sensitivity analysis, in which a higher IC50 represented a lower drug sensitivity. Compared with patients with low scores, those with high scores were more sensitive to drugs including A.443654 (Pan Akt inhibitor), AICAR (Acadesine), AUY922 (HSP90 inhibitor), AZD.0530 (Saracatinib, Src inhibitor), BI.2536 (PLK inhibitor), BI. D1870 (ATP competitive ribosomal S6 kinase inhibitor) (**Figures 10A–F**), whereas they were less sensitive to ABT. 263 (navitoclax), ABT. 888 (veliparib), AKT. inhibitor VIII, AS601245 (JNK inhibitor), ATRA (all-trans-retinoic acid), AZD6482 (PI3Kβ inhibitor) (**Figures 10G–L**). Therefore, this scoring model may have the potential to predict chemotherapy sensitivity in cervical cancer patients and guide chemotherapy regimen selection. However, current data relies on *in vitro* experiments, and further research is needed in the future to support this conclusion.

### 3.8 FOXJ1 inhibited the proliferation, migration, and invasion of cervical cancer cells through the regulation of cuproptosis

**Figure 11:**
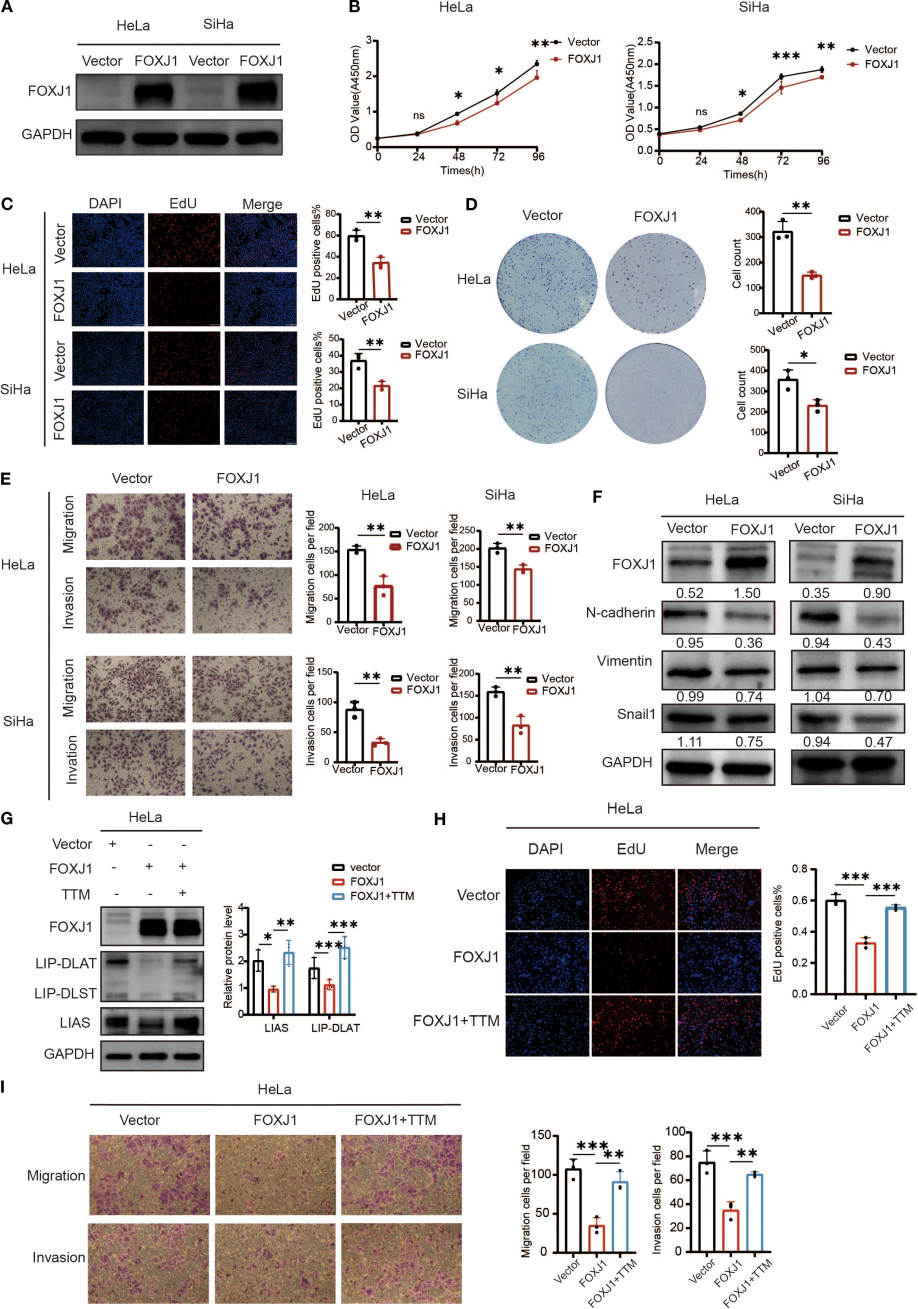
Overexpression of FOXJ1 decreased the proliferation, invasion, migration and the level of EMT process through the regulation of cuproptosis. **(A)** Western blotting showed the overexpression efficiency of FOXJ1 plasmid in HeLa and SiHa cells. **(B, C)** CCK-8 and EdU assays showing FOXJ1 attenuation of the proliferation of cervical cancer cells. **(D)** Colony formation assay showing reduced ability of forming colonies after overexpression of FOXJ1. **(E)** The migration and invasion abilities of cervical cancer cells determined by Transwell assay. **(F)** Overexpression of FOXJ1 reduced the protein levels of N-cadherin, Vimentin and Snail in cervical cancer cells. **(G)** Western blot analysis revealed that overexpression of FOXJ1 downregulated the expression of key cuproptosis-related proteins, LIAS and LIP-DLAT. This suppression was reversed upon treatment with the copper chelator TTM (40μM). **(H)** EdU assays demonstrated that FOXJ1 inhibits the proliferation of HeLa cells via cuproptosis. **(I)** Transwell assay experiments indicated that FOXJ1 suppresses the migration and invasion abilities of HeLa cells through the cuproptosis. Data are presented as the mean ± SD. *p < 0.05, **p < 0.01, *** *p* < 0.001.

To verify our previous results, we selected FOXJ1 and tested its effect on the growth of cervical cancer cells. We constructed an overexpression plasmid of FOXJ1 that was then transfected into HeLa and SiHa (**Figure 11A**). CCK-8 and EdU assays showed that FOXJ1 overexpression significantly inhibited cell proliferation capacity (**Figure 11B** and C). Furthermore, the colony formation assay showed that FOXJ1 overexpression significantly reduced colony formation ability of HeLa and SiHa cells (**Figure 11D**). The Transwell assay showed that the invasion and migration ability of cervical cancer cells decreased significantly after FOXJ1 over expression (**Figure 11E**). Epithelial-mesenchymal transition (EMT) was one of the main mechanisms responsible for tumor invasion and metastasis. To confirm whether FOXJ1 regulates invasion and metastasis through EMT, we further detected the regulatory role of FOXJ1 in the expression of EMT-related proteins. Western blotting analysis showed that overexpression of FOXJ1 dramatically reduced the expression of mesenchymal markers such as N-cadherin, vimentin, and Snail1 (**Figure 11F**). Thus, FOXJ1 could affect the invasion and migration of cervical cancer cells through the regulation of the EMT process.

To investigate the regulatory role of FOXJ1 in cuproptosis, Western blot analysis was conducted in cervical cancer cells. The results demonstrated that FOXJ1 overexpression significantly downregulated the protein expression of key cuproptosis markers, including LIP-DLAT and LIAS (**Figure 11G**). Notably, this suppressive effect was reversed upon concurrent treatment with the copper chelator TTM. EdU proliferation assays revealed that FOXJ1 overexpression markedly decreased cell proliferation, an effect that was abolished by TTM co-treatment (**Figure 11H**). Furthermore, Transwell migration/invasion assays showed that FOXJ1 overexpression significantly impaired the migratory and invasive capacities of HeLa cells, with TTM effectively rescuing these phenotypes (**Figure 11I**). Collectively, these findings indicate that FOXJ1 exerts tumor-suppressive effects in cervical cancer by inhibiting cuproptosis, thereby reducing cellular proliferation, invasion, and migration.

## 4 Discussion

Copper ions are involved in multiple important biochemical processes within cells, and an imbalance in copper ion homeostasis is associated with various diseases, including Menkes disease, Wilson disease, neurodegenerative diseases, and cancer (20, 21). Compared with healthy individuals, the serum copper levels of cervical cancer patients are significantly elevated. This suggests that excessive copper may be associated with the development and progression of cervical cancer (22). Copper-dependent cell death, also known as cuproptosis, is a newly discovered mode of cell death that provides new insights and targets for cancer treatment. In previous reports, researchers have constructed cuproptosis related models in cervical cancer and identified specific CRGs as prognostic markers for cervical cancer, which are involved in regulating multiple pathways including immune related pathways, TGF-beta signaling, Notch signaling, Hedgehog signaling pathway, and WNT signaling pathway (23). However, some of these studies focused on lncRNAs rather than CRGs; others heavily rely on the single step LASSO Cox method, which limits their applicability in variable screening, parameter optimization, interaction identification, and clinical practice. To overcome these limitations, we introduced a multi-level analysis framework. We first identified six genes associated with prognostic cuproptosis and their initial molecular subtypes. Then, we extracted differentially expressed genes (DEGs) between these subtypes and screened for 11 prognostic DEGs, achieving more refined patient stratification. Finally, we applied principal component analysis (PCA) to obtain DEG scores, capturing major transcriptome variations and achieving robust sample classification. The DEG score constructed based on it has better interpretability and generalization ability.

We comprehensively analyzed the characteristics of cuproptosis by mining and analyzing data from 334 cases of cervical cancer from TCGA and GEO databases. We analyzed 48 CRGs and selected 6 CRGs to construct a cuproptosis-related gene signature. Using this signature, the patients were divided into Cluster A and Cluster B. Compared with Cluster A, most cuproptosis genes (such as CCDC22, PDHA1, ARF1, and AQP1) in Cluster B were highly expressed, resulting in a better prognosis for patients with cervical cancer. CCDC22 is essential for copper ion homeostasis. It is involved in copper-dependent ATP7A trafficking between the trans Golgi network and vessels within the cell perimeter (24). PDHA1 (Pyruvate dehydrogenase E1 alpha 1) is an enzymatic component of the mitochondrial multienzyme complex and provides the primary link between glycolysis and the cycle of tricarboxylic acid (TCA) (25). ARF1 has a conserved role in the regulation of copper uptake in cultured mammalian cells (26). AQP1, a hydrodynamic transmembrane protein, primarily mediates the transport of water molecules and can also transport some cations across membranes (27). However, their crosstalk with cuproptosis is unknown in cervical cancer. Our results suggest the possible roles of CCDC22 and PDHA1 in the regulation of cuproptosis in cervical cancer, which deserves further investigation.

Mitochondrial respiration plays a crucial role in cuproptosis (28). Tsvetkov et al. found that hypoxia (1% O_2_) reduces sensitivity to cuproptosis by forcing cells to rely on glycolysis rather than oxidative phosphorylation (13). Consistently, our pathway enrichment analysis showed that the expression of cuproptosis genes was negatively associated with hypoxia and glycolysis. This suggested a close correlation between hypoxia, glycolysis, and cuproptosis in cervical cancer. Furthermore, the expression of cuproptosis genes was significantly correlated with the metabolism of glucose, galactose, fructose, and mannose, suggesting the essential role of cuproptosis in the regulation of glycometabolism in cervical cancer cells. Copper has been shown to directly stimulate endothelial cell migration and proliferation, as well as fibronectin synthesis, thus promoting angiogenesis (29). Consistently, our results indicated that cuproptosis genes were associated with smooth muscle cell contraction and EGFR signaling pathways in blood vessels. Considering the important role of antiangiogenic drugs such as bevacizumab and rituximab in the targeted treatment of cervical cancer (30), this result suggested the potential of targeting cuproptosis genes in the treatment of cervical cancer. Furthermore, we found that cuproptosis was closely related to many important signaling molecules such as TGF- β, TNF- α, target genes for E2F and MYC, MTORC1, EGFR, and SMAD. Given this potential role in the regulation of migration and growth of cervical cancer cells, cuproptosis may also be involved in various biological processes of cervical cancer and played an important role in cervical cancer.

It was noteworthy that our cuproptosis model showed good potential to predict the tumor microenvironment and guide tumor immunotherapy. Immune cells and stromal cells are the two main types of non-tumor components in the tumor microenvironment. Patients with high levels of immune cells and matrix components have a significantly better prognosis (31). In the present study, we found that patients with higher expression of CRGs had higher immune and stromal scores, indicating higher stromal components and infiltration of immune cells within the tumor. The cuproptosis gene-based signatures derived in this study could predict stromal components and immune cell infiltration of tumors. The infiltration of tumor-related immune cells and the expression of immune markers play a key role in the formation and treatment of tumors. In this study, we found that cuproptosis was positively correlated with different immune cell types such as activated B cells, activated CD8+T cells, myeloid derived suppressor cells, and macrophages. According to the score model derived using cuproptosis DEGs, the scores were positively correlated with activated B cells and activated CD8+T cells, and negatively correlated with neutrophils and Th2 helper T cells. Undoubtedly, CD8+T cells played an important role in antitumor cellular immunity (32). Therefore, high levels of cuproptosis suggested more infiltration of CD8+T cells and a better prognosis in patients. Pathway analysis showed that the cuproptosis model was closely related to the expression of chemokine ligands and receptors. Chemokines are involved in various cancer development processes, such as angiogenesis, tumor invasion and metastasis, tumor stemness, and immune cell infiltration (33). They are key determinants of disease progression and have a significant impact on the prognosis of the patient and the response to treatment. As a result of their important regulatory functions in immune-infiltrating cells, chemokine ligands and their receptors have become very powerful therapeutic targets. CXCL11 and CXCL14 are considered inhibitors of angiogenesis. The CXCL12/CXCR4 axis has been confirmed to regulate tumor metastasis in different tumors (34). Furthermore, CCR2 plays an important role in inducing the recruitment of monocytes and myeloid-derived suppressor cells (MDSCs) to tumors (35). And our results showed that scores were closely related to the expression of the aforementioned factors. Therefore, our model could effectively predict the tumor microenvironment.

As an emerging tumor immunotherapy, the US Food and Drug Association has approved multiple immune checkpoint inhibitors, such as anti CTLA-4 antibodies and PD-1/PD-L1 inhibitors for clinical use. The application of immune checkpoint inhibitors has received increasing attention in the treatment of patients with cervical cancer, and pembrolizumab had been approved for the treatment of recurrent or metastatic cervical cancer after first-line chemotherapy (36). In the score model of this study, patients with higher scores had higher levels of immune checkpoint expression, such as CTLA4, PDCD1, and TIGIT, suggesting that these patients were more likely to benefit from immunotherapy. It was interesting that CD274 was expressed to a lower degree in the higher DEG score group and we speculated that PD-L1 was highly expressed in tumor tissue in the lower DEG score group, which contributed to tumor immunoescape mechanisms and could lead to a poor prognosis. Therefore, the model we constructed could effectively predict the tumor microenvironment and the efficacy of cervical cancer tumor immunotherapy.

To further validate the reliability of the constructed cuproptosis model, we selected the FOXJ1 gene and validated its effect on the growth of cervical cancer cells. Forkhead box J1 (FOXJ1) belongs to the Fox gene family and has been shown to play a complex and crucial role in processes of development, organogenesis, regulation of the immune system, and progression of human malignancies (37). However, the role of FOXJ1 in cervical cancer remains unclear. Our findings showed that FOXJ1 overexpression in cervical cancer significantly inhibited the proliferation and colony formation capacity of cervical cancer cells and significantly weakened their invasion and migration ability. This process might be achieved by regulation of the EMT process. FOXJ1 significantly enhances the cuproptosis of cervical cancer cells, thereby effectively inhibiting their proliferation, invasion, and migration capabilities through the activation of this cell death pathway. This mechanism suggests that FOXJ1 may represent a potential therapeutic target for regulating the progression of cervical cancer. The results further confirmed the feasibility of tumor typing based on cuproptosis-associated genes.

Inevitably, our research has some limitations. Firstly, our study is based on retrospective retrieval data from TCGA and GEO databases. Due to the limited number of enrolled patients, the multivariate Cox analysis results of this model are not particularly robust. Further validation of the prognostic features of cuproptosis related genes is needed in large-scale, multicenter prospective cohort studies in the future. Secondly, the potential detailed mechanisms of cuproptosis and its related genes require further research in cervical cancer at both *in vivo* and *in vitro* levels. Thirdly, although FOXJ1 has been shown to regulate the growth of cervical cancer cells through cuproptosis, the *in vivo* validation and molecular mechanism exploration are still needed in the future.

## 5 Conclusion

New cell death mechanisms often present the potential for new targets for tumor treatment and promote personalized treatment strategies. In this study, we comprehensively analyzed the clinical and molecular characteristics of CRGs in cervical cancer and constructed a cuproptosis-related gene signature based on them. Our study revealed that cuproptosis was closely related to the prognosis of patients with cervical cancer, the activation/inhibition of cancer marker pathways, the tumor microenvironment, and the response to treatment, which represent a valuable resource and a rationale for subsequent research. Further research on cuproptosis in cervical cancer is expected to provide guidance for the development of personalized treatment plans for cervical cancer and to improve the stratification of patient prognosis in the future.

## Data Availability

The original contributions presented in the study are included in the article/**Supplementary Material**. Further inquiries can be directed to the corresponding author.

## Supplementary material

Supplementary Figure 1 Kaplan–Meier survival curves of CRGs could predict overall survival rate of patients with cervical cancer. The relationship between 48 CRGs and overall survival rate of the patients were analyzed using Kaplan-Meier analysis. Twenty-four genes were identified that could significantly predict the patient’s overall survival rate.
