# Supplementary figures and images for: Construction of a novel cuproptosis-related gene signature for predicting microenvironment, prognosis and therapeutic response in cervical cancer
Source: Front Oncol. 2025 Oct 10;15:1532772. doi: 10.3389/fonc.2025.1532772 (PMC12549250; doi:10.3389/fonc.2025.1532772)

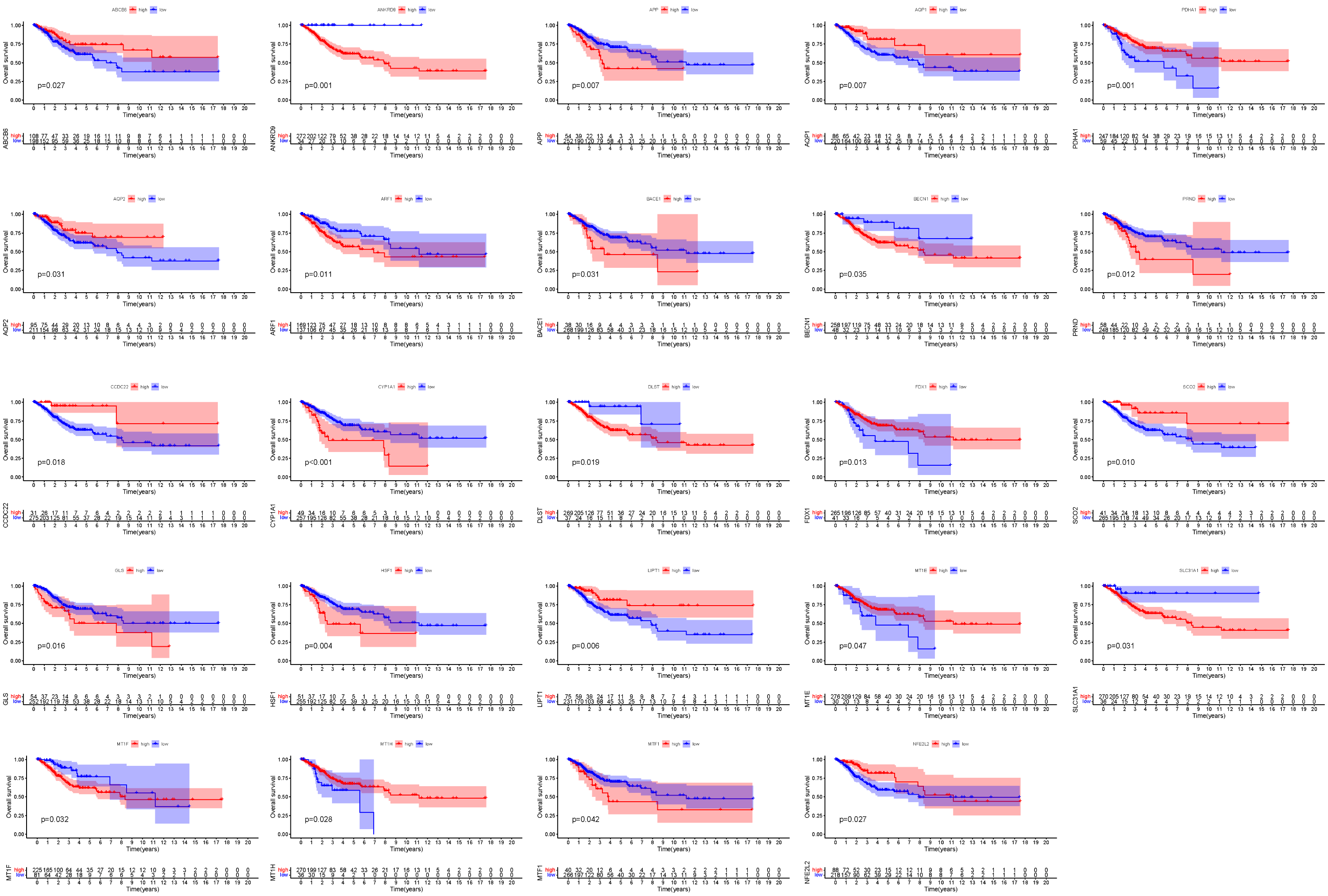

Supplement: Supplementary Figure 1 — Kaplan–Meier survival curves of CRGs could predict overall survival rate of patients with cervical cancer. The relationship between 48 CRGs and overall survival rate of the patients were analyzed using Kaplan-Meier analysis. Twenty-four genes were identified that could significantly predict the patient’s overall survival rate. [file Image1.tif]
